# Rare triad of periampullary carcinoid, duodenal gastrointestinal stromal tumor and plexiform neurofibroma at hepatic hilum in neurofibromatosis type 1: a case report

**DOI:** 10.1186/s12885-017-3567-z

**Published:** 2017-08-29

**Authors:** Nihed Abdessayed, Rahul Gupta, Sarra Mestiri, Ahlem Bdioui, Mounir Trimech, Moncef Mokni

**Affiliations:** 1 Department of pathology, Farhat Hached Hospital, Avenue Farhat Hached, 4000 Sousse, Tunisia; 2 Research Lab: transfer in technology in anatomic pathology (LR12SP08), Sousse, Tunisia; 3 Department of HPB surgery, CARE hospital, Hyderabad, India

**Keywords:** Case report, Neurofibromatosis, Neuroendocrine tumor, Gastrointestinal stromal tumor, Neurofibroma, Whipple’s operation

## Abstract

**Background:**

Neurofibromatosis type 1 is a relatively common inherited disorder. Patients with neurofibromatosis type 1 are at high risk of developing neurogenic, neuroendocrine and mesenchymal intra-abdominal tumors. Although coexistence of multiple tumors of different types is frequent in neurofibromatosis type 1, simultaneous occurrence of abdominal tumors of three types in very rare.

**Case presentation:**

A 66-year-old lady with neurofibromatosis type 1 presented with painless progressive jaundice for six months. Laboratory investigations revealed iron deficiency anemia and conjugated hyperbilirubinemia. Tumor markers were normal. Abdominal computed tomography showed a 3 × 2 cm heterogenous mass in the periampullary region with mild dilation of the common bile duct and another 2 × 1.7 cm mass in the fourth portion of the duodenum. Endoscopic biopsy confirmed the diagnosis of periampullary carcinoid. At surgery, multiple small nodules were detected at the hepatic hilum. Frozen section suggested them to be neurofibromas. Patient underwent pancreatoduodenectomy and had uneventful recovery with no recurrence at two months. Microscopic examination of the resected specimen confirmed presence of three tumors: periampullary well differentiated neuroendocrine tumor, gastrointestinal stromal tumor of the fourth part of duodenum and plexiform neurofibroma at the hepatic hilum.

**Conclusion:**

Patients of neurofibromatosis type 1 with abdominal symptoms should be treated with high index of clinical suspicion and thoroughly evaluated to rule out multiple tumors.

## Background

Neurofibromatosis type 1, also known as von Recklinghausen disease is an autosomal dominant disorder with the incidence of approximately one in 3000 births [1]. It occurs due to germline mutation of the neurofibromatosis type 1 gene located on chromosome 17 [2]. Neurofibromatosis type 1 gene encodes for a protein acting as a negative regulator of the ras proto-oncogene, which plays an important role in controlling cell growth. However, about 50% of the cases present with new mutations (de novo) without any family history of the disease [3].

Clinically, it is characterized by multiple cutaneous neurofibromas, café-au-lait spots, Lisch nodules (pigmented iris hamartomas) and axillary or inguinal freckling [4]. In addition to central and peripheral nervous system affection, patients with neurofibromatosis type 1 have a higher risk to develop benign or malignant tumors in other parts of the body [3]. Gastrointestinal tract involvement in neurofibromatosis type 1 is less frequent with the reported incidence of 10–25% [5, 6]. On the basis of histologic type, digestive tract neoplasms related to neurofibromatosis type 1 can be divided into five groups [7]: neurogenic neoplasms (neurofibroma, plexiform neurofibroma, ganglioneuroma), neuroendocrine tumors (NET) (carcinoid, pheochromocytoma, paraganglionoma), non-neurogenic mesenchymal tumors (gastrointestinal stromal tumor {GIST}, leiomyoma, leiomyosarcoma), embryonal tumors (rhabdomyosarcoma, neuroblastoma, Wilms tumor) and miscellaneous tumors (gastrointestinal adenocarcinoma). Due to variable penetrance, the patient may present with one or more of the above-mentioned tumors in synchronous or metachronous fashion. Although there are several reports of patients with synchronous presentation of abdominal tumors of two types, coexistence of three different tumors is very rare [8]. We report a rare triad of periampullary carcinoid, duodenal GIST and plexiform neurofibroma at hepatic hilum in a lady with neurofibromatosis type 1.

## Case presentation

**Fig. 1:**
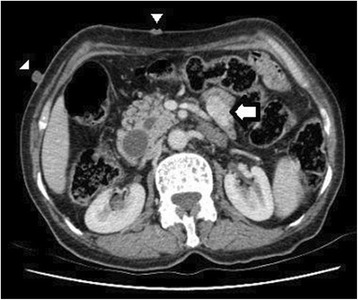
Contrast enhanced computed tomography of abdomen showing the duodenal gastrointestinal stromal tumor (arrow) and neurofibroma (arrowhead)

**Fig. 2:**
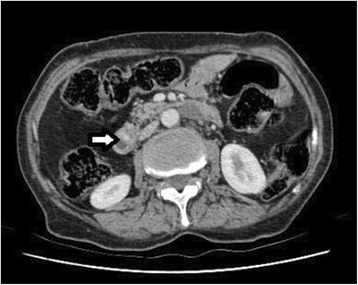
Contrast enhanced computed tomography of abdomen showing the small enhancing periampullary neuroendocrine tumor (arrow)

A 66-year-old lady with history of diabetes mellitus was referred for evaluation of painless progressive jaundice for six months associated with fatigue. Physical findings of café-au-lait spots, multiple neurofibromas over the trunk (Fig. 1) along with family history of first degree relative (sister) affected by neurofibromatosis type 1 confirmed the diagnosis of neurofibromatosis type 1 [9, 10]. Generalized icterus was noted however no abdominal lump was palpable. Laboratory analysis showed anemia (hemoglobin - 8.7 g/dl) and deranged liver function tests (total bilirubin – 246 umol/L, conjugated bilirubin – 132 umol/L, AST – 97 U/L, ALT – 70 U/L, GGT – 461 U/L, alkaline phosphatase – 659 U/L). Levels of carcinoembryonic antigen and carbohydrate antigen 19–9 were within normal range. Abdominal computed tomography showed a 3 × 2 cm lesion in the periampullary area of the duodenum showing heterogenous enhancement with mild dilation of the common bile duct and another 2 × 1.7 cm lesion in the fourth part of the duodenum (Figs. 1 and 2). Endoscopy showed a bulging mass at the site of major papilla. Biopsy was performed and histologic analysis revealed infiltration of lamina propria and muscularis mucosa with carcinoid cells that were strongly positive for synaptophysin and chromogranin. Urine 5-hydroxyindoleacetic acid levels were within normal limits. The patient underwent pancreatoduodenectomy. Intraoperatively, multiple small nodules were found on the hepatic hilum with the maximum size of approximately 4 cm. Thought to be metastatic lymph nodes, frozen section was performed which revealed spindle cell tumor. The patient had an uneventful postoperative recovery.

**Fig. 3:**
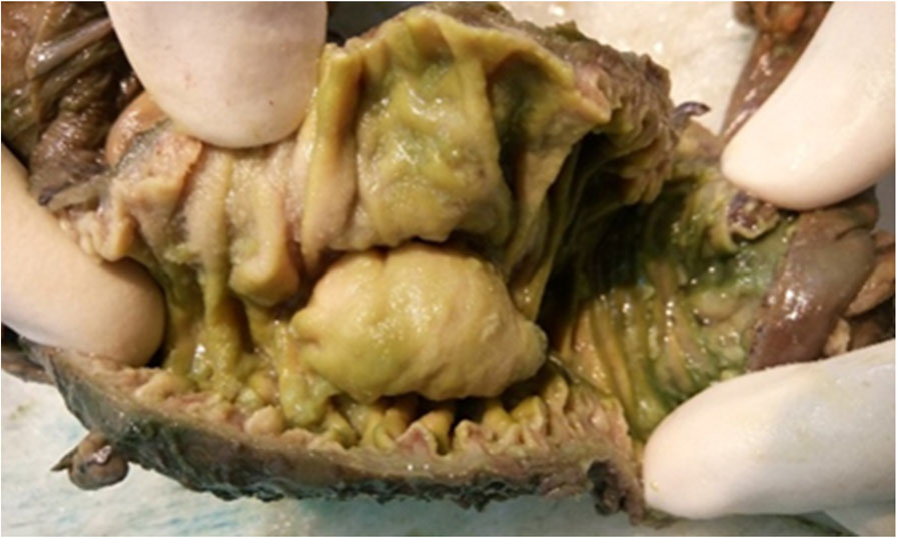
Macroscopic appearance of the periampullary tumor

**Fig. 4:**
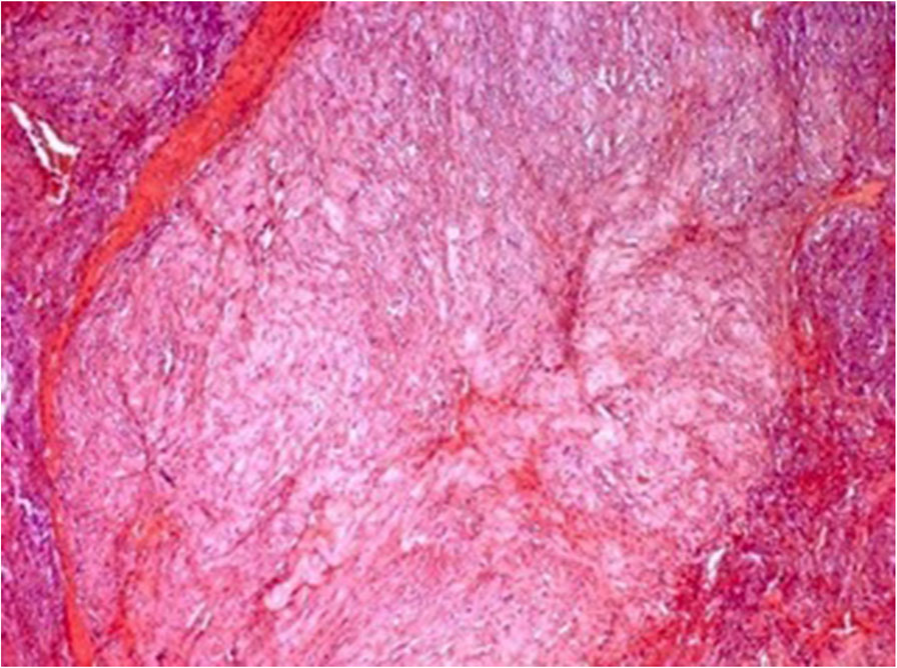
Microscopic appearance of the duodenal gastrointestinal stromal tumor showing skeinoid fibers (H&E × 200)

**Fig. 5:**
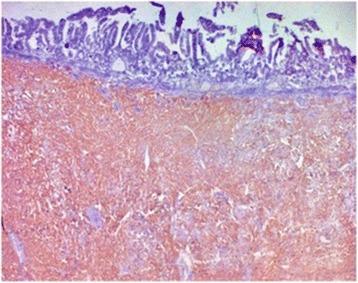
Duodenal GIST showing positive staining with CD117 (× 100)

**Fig. 6:**
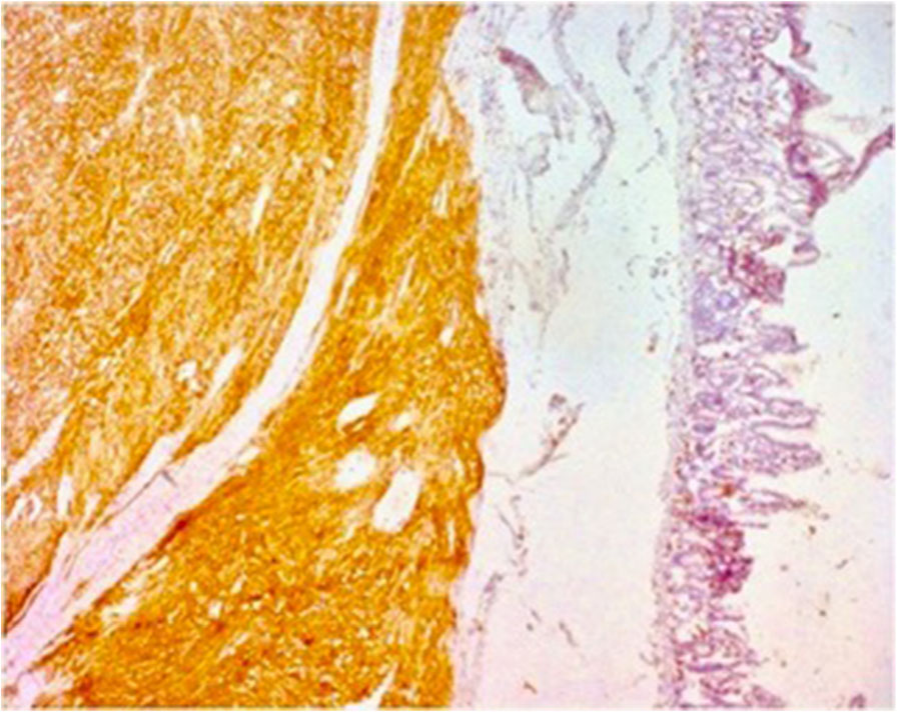
Duodenal GIST showing positive staining with DOG1 (× 100)

**Fig. 7:**
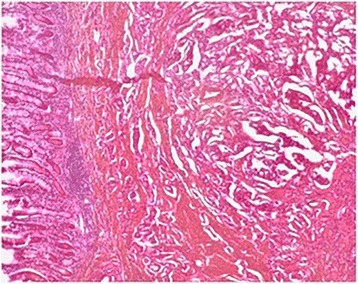
Microscopic appearance of the periampullary well differentiated neuroendocrine tumor (H&E × 100)

**Fig. 8:**
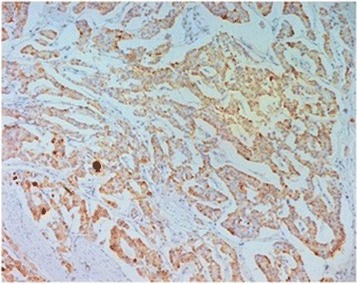
Positive staining of the tumor cells with chromogranin (× 200)

**Fig. 9:**
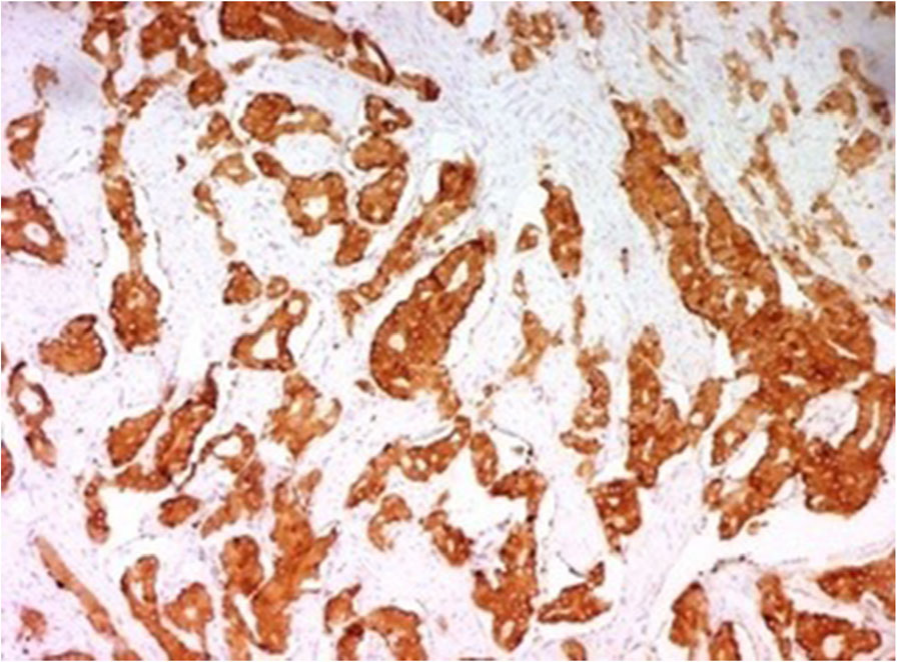
Positive staining of the tumor cells with synaptophysin (× 100)

The operative specimen (Fig. 3) showed a 3 × 2 × 2 cm non-ulcerated tumor located close to the ampulla of Vater. In addition, firm white-tan submucosal nodule sized 2 cm in greatest diameter was located in the fourth part of duodenum. Microscopically, the later was GIST, consisting of spindle cells arrange in a fascicular pattern without necrosis (Fig. 4). The nuclei were elongated without any significant pleomorphism. The cytoplasm was variably abundant with indistinct cell borders. Mitotic figures were infrequent. Immunohistochemical staining was strongly positive for CD34, CD117 and DOG1 supporting the diagnosis of GIST (Table 1) (Figs. 5 and 6). S100 stain was negative. Histopathological examination of periampullary tumor revealed well-differentiated endocrine tumor Grade 1 with strong immunohistochemical expression of chromogranin, synaptophysin and Ki67 < 1% (Figs. 7, 8 and 9). Also, CK7 and S100 were negative which ruled out adenocarcinoma and paraganglionoma respectively. Histologic examination of hepatic hilar nodules showed a spindle cell tumor arranged in fascicular pattern within fibrillar stroma. Nuclei were elongated, thin, without mitotic figures. Tumor cells were positive for S100 and were negative for CD34, CD117 and DOG1 suggestive of plexiform neurofibroma. All the resection margins were free of tumor. At two months of follow-up after surgery patient was doing well.

**Table 1:** Details about the clones, sources and dilutions of the various antibodies used for immunohistochemistry

Antibody	Clones	Source	Dilution
Synaptophysin	27G12	Novo Castra	1/100
CD117/c-Kit	Rb	Dako	1/400
Chromogranin A	5H7	Novo Castra	1/100
CD34	QEnd.10	Novo Castra	1/50
CK7	OV.TL	Dako	1/50
DOG-1	K9	Novo Castra	1/30
S100	Rb poly	Dako	1/400
Ki67	Mib-1	Dako	1/50

## Discussion and conclusions

Although abdominal tumors are more frequent in neurofibromatosis type 1 than general population, simultaneous existence of three different types of abdominal tumors is extremely rare [8]. To the best of our knowledge, coexistence of periampullary carcinoid, duodenal GIST and plexiform neurofibroma at hepatic hilum has not been described in English literature.

Most of the cases of NET are sporadic but familial forms can be seen as a part of inherited syndromes like multiple endocrine neoplasia type 1 syndrome, tuberous sclerosis or neurofibromatosis [11]. In fact, NETs are reported in about 1% of patients suffering from neurofibromatosis with the most common site being the periampullary region [3]. Histologically, NET comprise a large spectrum that ranges from well differentiated NET grade 1 and 2 to poorly differentiated ones or NE carcinomas and approximately 41% of those occurring in neurofibromatosis type 1 patients were well differentiated. Clinical symptoms are multiple and variable depending on tumor size, compression and spread. The most common presenting symptoms are jaundice (65%) and pain (31%) [11].

Biologically, the most common type of peri-ampullary NET in neurofibromatosis type 1 patients is somatostatinoma (40%) [12]. Diagnosis is made by a combination of radiological imaging, endoscopic ultrasound or endoscopy and measurement of 5-hydroxyindolectic acid and chromogranin [3]. Pancreaticoduodenectomy is the preferred treatment for well-differentiated ampullary carcinoid greater than 2 cm and for ampullary neuroendocrine carcinomas [12]. Local tumor excision can be considered for ampullary carcinoid less than 2 cm [12].

GIST is the most frequent mesenchymal tumors of the gastrointestinal tract in neurofibromatosis type 1 [13, 14]. The incidence in neurofibromatosis type 1 patients is nearly 45-fold higher than that in normal population [14]. The characteristic features of GIST in neurofibromatosis type 1 have been summarized in Table 2. Histologically, majority of tumors have spindle cell morphology with lower mitotic rate and Ki67 index [4, 14]. Surgery is the only treatment available as they are resistant to imatinib mesylate therapy due to lack of c-kit mutations [5]. The biological behavior is similar to that of GIST in non-neurofibromatosis type 1 patients [7].

**Table 2:** Comparison of characteristics of GIST in NF1 and sporadic cases

Characteristics	GIST in NF1	Sporadic GIST
Most common location	Small bowel	Stomach
Solitary or multiple	Multiple	Solitary
Association with other gastrointestinal tumors like carcinoid	Frequent	Rare
KIT or PDGFRA mutations	Absent	Present
Probable molecular pathogenetic mechanism	Activation of ras-MAP kinase cascade Mitotic recombination Loss of heterozygosity at 14q and 22q	Gain of function mutation of c-kit proto-oncogene
Response to Imatinib mesylate	Poor	Good

Plexiform neurofibroma is a one of the diagnostic features of neurofibromatosis type 1 [7]. They can be present anywhere in the body. Intra-abdominal plexiform neurofibromas are rare with less than 25 cases reported in literature [15]. They are most frequently located in the abdomino-pelvic wall or retroperitoneum [7, 15]. Plexiform neurofibroma involving the liver or periportal region comprise 2.3% of all abdominal plexiform neurofibromas [15–17]. Most of these tumors are incidentally detected during abdominal imaging or surgery as seen in our case [15]. In the present case, they were misinterpreted as metastatic lymph nodes. Rarely can they obstruct the main portal vein leading to portal hypertension [15]. These lesions are benign but have the potential to develop into malignant peripheral nerve sheath tumors [7, 15]. Because of the rarity of the disease, optimal timing and extent of surgery is not known. Complete surgical excision is often not feasible due to entrapment of the important vessels within the lesions [15–17].

In conclusion, patients with neurofibromatosis type 1 have high risk of harboring different intra-abdominal tumors simultaneously especially carcinoid and GISTs. High index of clinical suspicion, thorough preoperative and intra-operative evaluation is required to make correct diagnosis and provide appropriate treatment.

## Abbreviations

GIST: Gastrointestinal stromal tumor
NET: Neuroendocrine tumor
